# The effect of tertiary surveys on missed injuries in trauma: a systematic review

**DOI:** 10.1186/1757-7241-20-77

**Published:** 2012-11-29

**Authors:** Gerben B Keijzers, Georgios F Giannakopoulos, Chris Del Mar, Fred C Bakker, Leo MG Geeraedts

**Affiliations:** 1 Department of Emergency Medicine, Gold Coast Hospital, Gold Coast, Queensland, Australia; 2 Assistant Professor in Emergency Medicine, School of Medicine, Bond University, Gold Coast, Queensland, Australia; 3 Department of Surgery, VU University Medical Centre, Amsterdam, The Netherlands; 4 Professor of Public Health, School of Medicine, Bond University, Gold Coast, Queensland, Australia; 5 Department of Surgery, VU University Medical Centre, Amsterdam, The Netherlands

**Keywords:** Tertiary survey, Missed injury, Multiple trauma, Patient safety, Quality of care

## Abstract

**Background:**

Trauma tertiary surveys (TTS) are advocated to reduce the rate of missed injuries in hospitalized trauma patients. Moreover, the missed injury rate can be a quality indicator of trauma care performance. Current variation of the definition of missed injury restricts interpretation of the effect of the TTS and limits the use of missed injury for benchmarking. Only a few studies have specifically assessed the effect of the TTS on missed injury. We aimed to systematically appraise these studies using outcomes of two common definitions of missed injury rates and long-term health outcomes.

**Methods:**

A systematic review was performed. An electronic search (without language or publication restrictions) of the Cochrane Library, Medline and Ovid was used to identify studies assessing TTS with short-term measures of missed injuries and long-term health outcomes. ‘Missed injury’ was defined as either: Type I) any injury missed at primary and secondary survey and detected by the TTS; or Type II) any injury missed at primary and secondary survey *and* missed by the TTS, detected during hospital stay. Two authors independently selected studies. Risk of bias for observational studies was assessed using the Newcastle-Ottawa scale.

**Results:**

Ten observational studies met our inclusion criteria. None was randomized and none reported long-term health outcomes. Their risk of bias varied considerably. Nine studies assessed Type I missed injury and found an overall rate of 4.3%. A single study reported Type II missed injury with a rate of 1.5%. Three studies reported outcome data on missed injuries for both control and intervention cohorts, with two reporting an increase in Type I missed injuries (3% *vs.* 7%, *P*<0.01), and one a decrease in Type II missed injuries (2.4% vs. 1.5%, *P*=0.01).

**Conclusions:**

Overall Type I and Type II missed injury rates were 4.3% and 1.5%. Routine TTS performance increased Type I and reduced Type II missed injuries. However, evidence is sub-optimal: few observational studies, non-uniform outcome definitions and moderate risk of bias. Future studies should address these issues to allow for the use of missed injury rate as a quality indicator for trauma care performance and benchmarking.

## Background

Valid and reliable measures of trauma system performance are needed to guide improvement activities, benchmarking and research [1]. A common quality indicator in trauma care is *missed injury*, notwithstanding a systematic review finding limited validity and reliability [2].

Missed injuries occur in the time-critical and complex assessment of severely injured trauma patients in the Emergency Department (ED). Altered level of consciousness (from central nervous system injury, intoxication or sedation), distracting injury, or need for emergent surgery may impede adequate and detailed assessment of the patient. These initial examinations may therefore lead to injuries going undetected past the time when their management would avoid morbidity [3-14] or even mortality [5,7-9,15,16].

The trauma tertiary survey (TTS) is the proposed solution. It is an assessment undertaken after the episode of emergency care (including primary and secondary survey, emergency surgery and interventional radiology) and includes a comprehensive general physical re-examination and review of all investigations (diagnostic imaging and blood results) within 24 hours of admission [8,11,12], and repeated later when the patient is conscious, cooperative and mobilised [3,8,13].

*Missed injury* is most commonly defined as an injury missed at initial assessment up to 24 hours (including both primary and secondary survey and emergency intervention) [3,12,13]. With the TTS becoming part of standard trauma care, a second definition refers to injuries that were missed *despite* TTS performance [11]. Clearly then, the impact of a TTS on missed injuries will depend on which definition of missed injuries is used. An increase in *detected* injuries would be expected by the first definition, but a decrease of *missed* injuries by the second.

The TTS should be a useful tool for missed injury benchmarking. However, any benefits from performing routine TTS might be outweighed by excessive use of resources, or over-diagnosis (in which a TTS-identified injury has little or no effect on clinically relevant, long-term outcomes) [17]. Although the TTS should, by simple intuition, improve trauma care, we set out to test this empirically by systematically reviewing the literature. By doing so, we expect to facilitate the use of missed injury as a useful quality indicator in the future.

### Aims

The aim of this study was to systematically review the literature to determine the effect of the TTS in hospitalized trauma patients on both types of missed injury rates (Type I - missed at initial management, but detected by TTS; Type II - missed at initial management *and* by TTS, detected during hospital stay) and long-term health outcomes.

## Methods

### Study eligibility

We considered any study assessing a TTS using randomized or quasi-randomized trials, observational studies such as cohort, case–control and before-and-after design studies. Subjects were trauma patients admitted to any hospital, with no limits regarding age, gender, or severity of trauma.

We included any study that used the TTS as an intervention alone or as part of a larger intervention (such as a change in hospital trauma system). The TTS was defined as a review of the admitted patient within 24 hours (or after regaining consciousness) and included at least a repeated full physical examination.

The primary outcome was missed injury, defined as Type I) any injury missed at initial management (primary and secondary survey and emergency intervention) and detected by TTS, or Type II) any injury missed at initial management *and* TTS, detected during hospital stay. The missed injury rate was the proportion of patients with a missed injury within the study population. Secondary outcomes included long-term health outcomes including rates of injuries detected after hospital discharge and ability to return to pre-injury functional status. Eligible studies had to include either the primary or secondary outcome.

### Search strategy and information sources

Relevant studies were identified using electronic searches of MEDLINE (1966 to December 2010) and OVID (1980 to December 2010) and the Cochrane Library Central Registry of controlled trials, without language restrictions. The following key words were used to conduct the search: tertiary survey, trauma survey, traumatology, diagnostic errors, delayed diagnosis, missed diagnosis, missed injury, prognosis and long-term outcomes. The full search strategy is contained in Additional file 1.

### Selection of studies

Two reviewers (GK and GG) independently assessed all titles and abstracts for potential relevant articles, with any disagreement adjudicated by a third reviewer (LG). We retrieved the full-text article of any reference that appeared to meet the inclusion criteria. The eligibility of the full-text articles was assessed against the criteria of a standardized form.

### Data extraction and management

The following data were extracted from the studies: title, year of publication, country of study, study design, number of participants, age and gender of participants, injury severity score (median ISS, proportion with ISS>15), mechanism of trauma (blunt *vs.* penetrating), presence of an altered level of consciousness and admission to intensive care unit (ICU). The outcome parameters on missed injury rates and long-term outcomes were collected when available. Authors were contacted in order to obtain missing data. We attempted meta-analysis to quantify and summarize results, but due to the inherent bias of the studies and the extent of the heterogeneity [18,19], meta-analysis was deemed invalid and hence not reported. Primary and secondary outcomes were all proportions. Results of studies were pooled using simple weighted averages. Chi-square test was used to test for differences in proportions.

Since we anticipated possible differences in outcomes for certain demographic groups, we defined potential subgroups for analysis *a priori*, which included; age, gender, ISS (ISS>15), injury mechanism (blunt *vs*. penetrating), altered level of consciousness and ICU admission.

### Assessment of risk of bias

Two of us (GK and GG) independently used the Newcastle-Ottawa Scale [20] to assess the quality of non-randomized observational studies (Additional file 2). We classified studies to either low, moderate or high risk of bias if there were respectively up to 1, 2–3 or >3 inadequate items.

This systematic review was conducted to conform to the PRISMA standard (http://www.prisma-statement.org).

## Results

**Figure 1:**
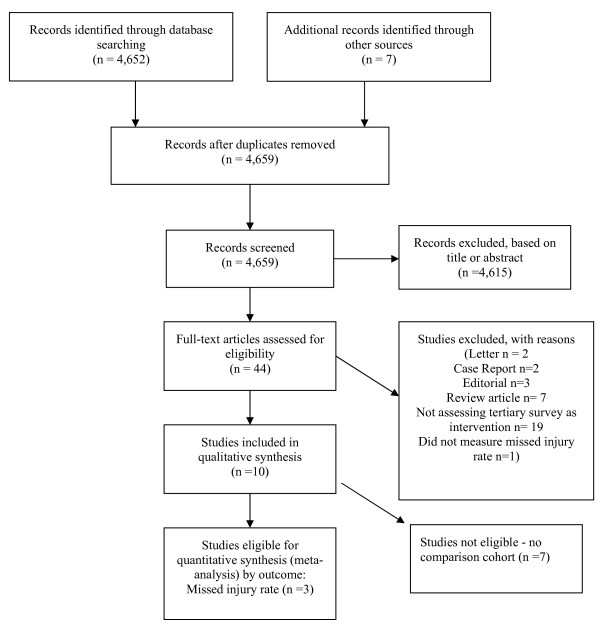
Selection of studies.

Our search identified a total of 4,659 of potentially relevant references. We discarded 4,615 after examining their Title or Abstract. The full-text articles of the remaining 44 were retrieved: 10 studies [3,11-13,21-25] were included in the review, (of which three were suitable for meta-analysis, Figure 1; Table 1). None were randomized or quasi-randomized trials, that is, all 10 included studies were observational, (seven prospective cohort studies [8,12,13,22-25]; one prospective cohort study with historical comparison [1]; and two cohort studies with a before-and-after design [11,21]).

**Table 1:** Description of included studies

**Author, year, origin**	**Population (number and description)**	**Population characteristics (Age, Gender, Mechanism, ISS)**	**Intervention**	**Outcome measure**	**Study design**
Enderson, 1990, Tennessee, USA	399 admitted trauma patients	Age>15 yrs: 86%	TTS as part of trauma admission form, conducted within 24–48 hours after patient stabilization	Missed injuries – defined as detected as a result of TTS. (Type I)	Prospective cohort study – comparing with historical summary data
		Gender: N/A			
		Mechanism: 89%			
		Blunt Mean ISS: 21			
Biffl, 2003	All admitted trauma patients.	Mean Age: 45.3 vs. 44.5 yrs	Implementation of formal TTS, using standardized form and TTS policy. TS within 24 hours and after ICU discharge	Missed injury rate – defined as injuries detected after 24 hours admission or injuries missed by TTS. (Type II)	Cohort study with before-and-after design
Rhode Island, USA	Before: 3,412	Gender: 63% vs. 64% Male			
	After: 3,442	Mechanism: N/A			
		Mean ISS: 10.7 vs. 10.7			
Vles, 2003	All (3,879) admitted trauma patients	Age: N/A	Use of standard trauma forms, TTS and review of radiology within 24 hours	Missed injury rate – Any injury missed on primary and secondary survey. (Type I)	Prospective cohort study
The Netherlands		Gender: N/A			
		Mechanism: N/A			
		ISS>16: 1.2%			
Hoff, 2004	432 admitted trauma patients	Age: N/A	Formal radiology rounds as part of TTS	Missed injury or ‘new diagnosis’ as result of radiology rounds with trauma surgeons. (Type I)	Prospective cohort study
Pennsylvania, USA		Gender: N/A			
		Mechanism: N/A			
		ISS: N/A			
Soundappan, 2004	76 children admitted with ISS>9	Mean Age: 8.5 yrs	TTS performed using standardized from by trauma fellow on day after admission and after extubation	Missed injury rate – Any injury missed on primary and secondary survey. (Type I)	Prospective cohort study
Sydney, Australia		Gender: 66% Male			
		Mechanism: 100% Blunt			
		Mean ISS: 15			
Howard, 2006	90 admitted trauma patients	Age: N/A	TTS performed using standardized from by single clinician within 24 hours	Missed injury rate – Any injury detected on the TTS. (Type I)	Prospective cohort study
Indianapolis, USA		Gender: 74% Male			
		Mechanism: N/A			
		ISS: N/A			
Okello, 2007	403 admitted trauma patients	Mean Age: 29 yrs	Daily physical examination up to 30 days, including TTS in first 24 hours	Missed Injury – unclear definition – implied as injury detected after primary and secondary survey. (Type I)	Prospective cohort study
Uganda		Gender: 82% Male			
		Mechanism: 91% Blunt			
		ISS: N/A			
Janjua, 2008	206 admitted trauma patients	Mean Age: 35 yrs	TTS performed by trauma fellow within 24 hours and after regaining consciousness	Missed injury rate – Any injury missed on primary and secondary survey and operating room. (Type I)	Prospective cohort study
Sydney, Australia		Gender: 75% Male			
		Mechanism: 91-100% Blunt			
		ISS: N/A			
Ursic, 2009	All admitted trauma patients.	Mean Age: 43.4 vs. 44.4 yrs Gender: 69.4% vs 68.9% Male Mechanism:94.3 vs. 94.4% Blunt ISS>15: 26% vs 31%	Implementation of a dedicated trauma service, which included a formalised TTS	Mortality and Length of Hospital stay. Missed injury – not in article -data retrieved via author communication - any injury missed at primary and secondary survey. (Type I)	Cohort study with before-and-after design
Sydney, Australia	Before: 981				
	After: 1,006				
Huynh, 2010	5,143 admitted trauma patients	Mean Age: 36.2 yrs	Mid level providers performed TTS using a form within 48 hours. This was reviewed by trauma surgeon	Missed injury – defined as detected at TTS. (Type I)	Prospective cohort study
North Carolina, USA		Gender: 71% Male			
		Mechanism: 85% Blunt			
		Mean ISS: 14.2			

*ISS* Injury Severity Score, *TTS* Tertiary Survey, *USA* United States of America.

The risk of bias was *low* for two studies [11,21] and *moderate* in the remaining eight (Additional file 2). The selection of participants in all studies was by consecutively admitted trauma patients, ensuring appropriate representativeness and minimizing selection bias. All ten studies used either medical records or a special form to determine whether a TTS had occurred. Seven studies did not have a comparison cohort (i.e. a cohort without TTS performed), increasing their risk of bias [8,12,13,22-25]. There was insufficient information to assess the comparability section of the Newcastle-Ottawa scale for eight studies. The remaining two studies [11,21] had comparable demographics for exposed and non-exposed cohorts. One study had a higher admission rate to the trauma ICU in the cohort receiving the TTS compared to those in the cohort who did not (30% vs. 20% admission rate) [11].

### Missed injury rate – in patients receiving TTS

Nine [3,12,13,21-25] of the included studies used Type I missed injury as outcome (missed at primary and secondary survey and detected by TTS), with the remaining single other study [11] utilizing Type II missed injury (injury missed by both initial assessment *and* the TTS).

### Type I missed injury rate – in patients receiving TTS

The overall Type I missed injury rate in cohorts with a TTS conducted was 4.3% (Table 2). The two largest studies [12,23] had similar Type I missed injury rates (1.3% and 1.6%, respectively), while a medium-sized study [21] had a Type I missed injury rate of 6.2% (unpublished data provided by author). The remaining studies reported Type I missed injury rates varying from 9.3 to 19.3%, with one small study [8] being an outlier (65%). In this study, a dedicated trauma surgery fellow aware of the purpose of the study performed all tertiary surveys.

**Table 2:** Outcomes – Type I missed injury rates

	**PRE TTS implementation**			**POST TTS implementation**		
	***Missed injuries***	***Study population (N)***	***Missed injury rate (%)***	***Patients with Missed injuries (N)***	***Study population (N)***	***Missed injury rate (%)***
Enderson, 1990		N/A	2.0	37	399	9.27
Vles, 2003				49	3,879	1.26
Hoff, 2004				42	432	9.72
Soundappan, 2004				12	76	15.8
Howard, 2006				12	90	13.3
Okello, 2007				78	403	19.4
Janjua, 2008				134	206	65.0
Ursic, 2009	35	981	3.57	62	1,006	6.16
Huynh, 2010				80	5,143	1.56
**Overall**	**35**	**981**	**3.57**	**506**	**11,634**	**4.35**

Injuries missed at initial assessment, detected by TTS.

TTS Tertiary Survey, N/A not avaible – a similar population size is assumed (N=399).

### Type II missed injury rate – in patients receiving TTS

Only one study reported Type II missed injury, with a rate of 1.51% after TTS introduction [11] Table 3.

**Table 3:** Outcomes – Type II missed injury rates

	**PRE Tertiary survey implementation**			**POST Tertiary survey implementation**		
	***Missed injuries***	***Study population (N)***	***Missed injury rate (%)***	***Missed injuries***	***Study population (N)***	***Missed injury rate (%)***
Biffl, 2003	81	3,412	2.37	52	3,442	1.51
**Overall**	**81**	**3,412**	**2.37**	**52**	**3,442**	**1.51**

Injuries missed at initial assessement *and* by TTS, detected in-hospital.

*TTS* Tertiary Survey.

### Type I and II missed injury rate – before and after introduction of TTS

Two studies [3,21] compared Type I missed injury and one study [11] compared Type II missed injury using before and after studies. Type I missed injuries increased as a result of the TTS (3% vs. 7%, *P* < 0.01), and type II missed injuries decreased (2.4% vs. 1.5%, *P* = 0.01).

### Clinical relevance of missed injuries

Table 4 summarizes the anatomical areas of missed injury, change in management and resultant morbidity or mortality as reported by the individual studies. There was a large variation in anatomical distribution of missed injuries, with orthopaedic extremity injuries, spine related injuries and facial injuries most commonly reported. The proportion of patients with missed injury requiring surgical intervention varied between 10-30%, which equates to 0.1-5% of the total population of these studies [3,8,12,13,22,23]. Only two deaths were reported specifically as a result of a missed injury [8].

**Table 4:** Description of missed injuries

**Author, year, origin, N**	***(N)*with MI**	**Area involved**	**%**	***(N)*with clinically significant MI**	**Description of change in management**	**Mortality and morbidity**
Enderson, 1990, Tennessee, USA N=399	*36*	MSK	51	*7*	OT, N= 7	Nil deaths
		Spinal	12		(MSK N=3, Facial N=1, Abdomen N=3)	Stroke, N=1
		Facial	5			
		Thoracic	12			
		Abdominal	15			
		Vascular	5			
Biffl, 2003,	*81 vs. 52*	MSK	32 vs. 46	*Not reported*	Not reported	Not reported
Rhode Island, USA		Spinal	29 vs. 24			
Pre TTS: N= 3412 vs. Post TTS: 3442		Abdominal	17 vs. 18			
		Brain	10 vs. 6			
		Pelvic	5 vs. 0			
		Vascular	3 vs. 2			
		Diaphragm	3 vs. 0			
Vles, 2003,	*49*	Chest	33	*22*	OT, N=12	Morbidity unspecified, N=3
The Netherlands N=3879		MSK	27		(Chest N=1, MSK N=4, Facial N=5, Other N=2)	
		Skull	7		ICC, N=2	
		Facial	13		Cast, N=6	
		C-Spine	7			
		Other	10		Halo/brace, N=2	
Hoff, 2004	*42*	Extremities	45	*19*	OT, N=4 (not specified)	Not reported
Pennsylvania, USA		Spine	21		Cast, N=7	
N=432		Chest	15		Transfer, N=1	
		Pelvis/proximal skeleton	19		Change in advice, N=6, Home equipment, N=1	
Soundappan, 2004	*12*	Head/face	33	*1*	OT, N=1 (not specified)	Nil deaths
Sydney, Australia		Spine	17			Prolonged LOS, N=4
N=76		Extremities	50			Delay in mobilisation, N=4
Howard, 2006,	*13*	Extremities	70	*Not reported*	Not reported	Not reported
Indianapolis, USA		Face	12			
N=90		Spine	12			
		Chest	6			
Okello, 2007,	*76*	Head and neck	24	*Not reported*	Not reported	Not reported
Uganda		Face	8			Mulivariate regression shows higher morbidity and longer LOS in patients with MI compared to patients without MI. This may not reflect causality.
N=403		Thorax	11			
		Abdomen/pelvis	20			
		Extremities	26			
Janjua, 2008	*134*	MSK	40	*30*	OT, N=11 (Orthopedic n=3, Laparatomy N=7,	Death 1.5% (N=2: C1 fracture; epidural hematoma)
Sydney, Australia		STI	36		Thoracotomy N=1)	Complications 8% (peritonitis N=4 after missed hollow viscus injury)
		Abdomen	6		Laceration repair, N=2	
N=206		Nerve injury	9		Embolisation, N=1	
		(Hemo-) Pneumothorax	5		Not specified, N=17	
Ursic, 2009	*35 vs 62*	Not reported		*Not reported*	Not reported	Mortality
Sydney, Australia						Pre 3.5% vs. post 2.5%
Pre TTS: N=981 vs. Post TTS= 1006						
Huynh, 2010	*80*	Orthopedic	60	*31*	OT, N=7 (Orthopedic N=4, Facial N=2, Spinal N=1)	Not reported
North Carolina, USA		Facial/plastics/dental	21		Cast, N=24	
N=5143		Neurosurgical	16			
		Ophthalmology	3			

Description of missed injuries. Anatomical area, clinically significant missed injuries, change in management and mortality and morbidity.

*MI* Missed Injury, *OT* operating Theatre, *ISS* Injury Severity Score, *TS* Tertiary Survey, *USA* United States of America, MSK musculoskeletal. Not all columns add up to 100% due to low proportions not being reported.

*TTS* Tertiary Trauma Survey.

### Subgroup analyses

Only one study investigated a difference in missed injury rates after introduction of a TTS for any of the pre-defined subgroups [11]. It reported a decrease in Type II missed injuries in patients admitted to a trauma intensive care unit (5.7% vs. 3.4%, *P* < 0.05). Another reported a paediatric trauma population (Type I missed injury rate: 15.8%), but did not specifically investigate age as a factor of interest [13]. A third study reported a lower mortality trend (5.4% vs. 4.1%, *P* = 0.17) associated with a higher detection of Type I missed injuries by TTS, introduced as part of a trauma service (3.6% vs. 6.2%, *P* < 0.01, via author communication) [21].

No other included study assessed differences in (any type of) missed injury rate after introduction of a TTS for the other pre-defined subgroups (age, gender, ISS, mechanism of injury or altered level of consciousness).

### Long-term health outcomes

No studies that assessed the effect of the TTS on long-term health outcome were identified.

### Completeness of data

Data for the two studies included in the analysis of the systematic review (using missed injury rate at initial assessment, detected by TTS) were not complete in the original publications. One quoted a historical *overall* missed injury rate of 2% (without data on sample size) [3] and another did not report missed injury data in the published manuscript [21]: we obtained this information by writing to the authors directly.

## Discussion

This systematic review found empirical evidence that the TTS improves trauma care by increasing Type I missed injuries and reducing Type II missed injuries.

### Limitations

Our findings were based on weak evidence: there were no relevant randomized studies, so only observational studies with their inherent risk of bias were available. Meta-analysis was attempted, but due to few studies being eligible and being prone to bias as well as substantial heterogeneity, we have not reported this. We were unable to assess the effect of TTSs on morbidity, so any improvement in patient outcomes consequent on reduction in missed injuries has to be inferred.

Other shortcomings included variation in the trauma patient populations (one study including paediatric trauma patients only [13]); geography (with one study conducted in Uganda [24] where trauma patterns and trauma care may be different from those of the other studies); and in the intervention (differently defined in two studies - one study [22] aimed to decrease missed injuries by formalizing the radiology review component of the TTS, while another [21] assessed the effect of implementing a complete trauma service, of which a TTS was a component).

The definition of missed injury varied between studies: one study defined missed injury as any injury that escaped detection at time of the TTS [11], (i.e. Type II). The other nine studies, including two before-and-after studies, used the more commonly used *‘any injury missed by primary and secondary survey, and detected as a result of the TTS’* (i.e. Type I), which really represents a delayed diagnosis (or increase in injury detection). For example, in one study [21] the TTS was associated with increased missed injury rate, but reduced mortality. This seems counter-intuitive until one realises this is related to Type I missed injuries, leading to more frequent detection at 24 hours by TTS.

This systematic review highlights several issues. Firstly *missed injury* needs a consistent, clear and expanded definition to facilitate future research and provide a tool for benchmarking. The use of these differences in definition precludes overall comparison of studies and need to be made explicit in order to legitimately compare studies. We propose a classification of missed injuries (Table 5). It is likely that the third group in this proposed classification (Type III: missed injury detected after hospital discharge) has been under-reported, since there are no published data.

**Table 5:** Missed injury classification

**Missed injury type**	**Description**
***Type I***	***Before TTS or as result of TTS:***
	Injury missed at initial assessment (primary and secondary survey and emergency intervention), but detected within 24 hours, before or through formal TTS (i.e. delayed diagnosis at 24 hours)
	(Injury missed at initial assessment)
***Type II***	***After TTS, during hospital stay:***
	Injury missed by TTS, detected in hospital after 24 hours.
	(Injury missed at initial assessment *and* TTS)
***Type III***	***After TTS, after hospital discharge:***
	Injury missed during hospital stay including TTS, detected after hospital discharge.
	(Injury missed at initial assessment *and* TTS *and* hospital stay)

Secondly, for the nine studies using the Type I missed injury definition, a mean injury detection rate of 4.3% was found. This can be used as a yardstick to compare future studies assessing Type I missed injuries. This may yet be an over-estimation of Type I missed injuries (or rather, delayed diagnoses), since the reported missed injury rate was approximately 1.5% in the two larger studies that together included more than 9,000 subjects and where investigator bias would have been minimal.

Furthermore, we summarized the anatomical distribution of missed injuries and how this changed management as reported by the individual studies (Table 4). This relates to the clinical relevance of these injuries, since it would be reasonable to expect at least delayed recovery or even prolonged morbidity without these interventions. Very few deaths as a result of a missed injury were reported and need for a change in management in the form of surgical intervention was variable or not reported. None of the studies included in this systematic review pre-defined clinical significance in the design of the study. Three studies discussed clinical relevance of missed injuries, with the common denominator being whether the missed injury would have lead to morbidity or mortality as judged by expert opinion (Table 6). Interestingly, one study defined a clinically significant missed injury as *any* change in management, including ordering further imaging [22]. We did not pre-define *clinically relevant* missed injury as an outcome of this systematic review. The available data suggests that the current literature does not have a widely agreed definition for clinically significant missed injury, evidenced by the variable reporting and outcomes. A more consistent and reproducible approach to this issue is warranted. Very few studies related the TTS to morbidity and mortality and as such we cannot comment on the effect the TTS had on patient outcomes.

**Table 6:** Definitions of clinically significant missed injury amongst included studies

**Author**	**Description**
Hoff et al.	*Level 1* - Missed injury would likely lead to morbidity/mortality
	*Level 2*- Missed injury alters care in hospital (including additional imaging)
Vles et al.	Any missed injury that leads to change in treatment resulting from the detection of the missed injury
Huynh et al.	Clinically significant missed injuries are injuries that are judged as such by the trauma attending and required intervention

Lastly, we found no reporting of long-term outcomes after TTS. Studies have reported long-term outcomes for the multiple injured patients [26] or subgroups of patients with specific injuries [27], but not the effect of the TTS *per se*.

## Conclusions

In cohorts with a TTS conducted, the Type I missed injury rate was 4.3% and Type II missed injury rate was 1.5%. The TTS increased Type I missed injuries (or injury detection by TTS), and decreased Type II missed injuries (less injuries missed by TTS). This is based on few studies with risk of bias, and as such the clinical effect of TTS is not fully known. This review emphasizes the lack of studies reporting long-term outcomes after a TTS. This may be due to assumed benefits of the TTS. Quantifying the actual effect on longer-term health outcomes, including missed injuries after hospital discharge, may support a more structured and widespread use of the TTS. Future studies using consistent outcome definitions are warranted to allow for the use of missed injury rate as a quality indicator for trauma care performance and benchmarking.

## Competing interests

The authors declare that they have no competing interests.

## Authors’ contributions

Design and concept (all authors), literature search (GK and GG), data extraction (GK and GG), data adjudication (LG), data analysis (GK), data interpretation (GK, LG, CdM), manuscript drafts and approval of final manuscript (All authors), responsibility for paper as a whole (GK). All authors read and approved the final manuscript.

## Supplementary Material

Additional file 1 Complete Search Strategy. Click here for file

Additional file 2 **Newcastle-Ottawa Scale for cohort studies**^**20**^**.** Click here for file
