# Supplementary material for: The effect of tertiary surveys on missed injuries in trauma: a systematic review
Source: Scand J Trauma Resusc Emerg Med. 2012 Nov 29;20:77. doi: 10.1186/1757-7241-20-77 (PMC3546883; doi:10.1186/1757-7241-20-77)
Supplement: Additional file 1 — Complete Search Strategy. [file 1757-7241-20-77-S1.doc]

Appendix 1 – Complete Search Strategy

| 1. exp Diagnostic Errors/  2. (missed adj2 diagnosis).mp. [mp=title, original title, abstract, name of substance word, subject heading word, unique identifier]  3. exp "Outcome and Process Assessment (Health Care)"/  4. (missed adj1 injur$).mp. [mp=title, original title, abstract, name of substance word, subject heading word, unique identifier]  5. (delayed adj1 diagnosis).mp. [mp=title, original title, abstract, name of substance word, subject heading word, unique identifier]  6. (prognostic adj1 outcome$).mp. [mp=title, original title, abstract, name of substance word, subject heading word, unique identifier]  7. exp treatment outcome/  8. (long adj1 term adj1 outcome$).mp. [mp=title, original title, abstract, name of substance word, subject heading word, unique identifier]  9. 1 or 2 or 3 or 4 or 5 or 6 or 7 or 8  10. exp Emergency Service, Hospital/  11. exp Emergency Medical Services/  12. Emergencies/  13. exp Emergency Treatment/  14. exp Critical Care/  15. Traumatology/  16. exp "Wounds and Injuries"/  17. trauma.mp. [mp=title, original title, abstract, name of substance word, subject heading word, unique identifier]  18. 10 or 11 or 12 or 13 or 14 or 15 or 16 or 17  19. Medical History Taking/  20. data collection/ or health surveys/ or questionnaires/  21. (trauma adj1 system$).mp. [mp=title, original title, abstract, name of substance word, subject heading word, unique identifier]  22. (tertiary adj1 survey$).mp. [mp=title, original title, abstract, name of substance word, subject heading word, unique identifier]  23. tertiary trauma survey$.mp. [mp=title, original title, abstract, name of substance word, subject heading word, unique identifier]  24. (trauma adj1 survey$).mp. [mp=title, original title, abstract, name of substance word, subject heading word, unique identifier]  25. (primary adj1 survey$).mp. [mp=title, original title, abstract, name of substance word, subject heading word, unique identifier]  26. (secondary adj1 survey$).mp. [mp=title, original title, abstract, name of substance word, subject heading word, unique identifier]  27. survey$.mp. [mp=title, original title, abstract, name of substance word, subject heading word, unique identifier]  28. trauma severity indices/ or injury severity score/  29. 19 or 20 or 21 or 22 or 23 or 24 or 25 or 26 or 27 or 28  30. 9 and 18 and 29  31. 9 or 18 or 29 |
| --- |
