# Supplementary material for: The effect of tertiary surveys on missed injuries in trauma: a systematic review
Source: Scand J Trauma Resusc Emerg Med. 2012 Nov 29;20:77. doi: 10.1186/1757-7241-20-77 (PMC3546883; doi:10.1186/1757-7241-20-77)
Supplement: Additional file 2 — Newcastle-Ottawa Scale for cohort studies20. [file 1757-7241-20-77-S2.doc]

Appendix 2. Newcastle-Ottawa Scale for cohort studies^20^

| **Selection**  1) Representativeness of the exposed cohort  a) truly representative of the average admitted trauma patient *  b) somewhat representative of the average admitted trauma patient *  c) selected group of users e.g. nurses, volunteers  d) no description of the derivation of the cohort  2) Selection of the non exposed cohort  a) drawn from the same community as the exposed cohort*  b) drawn from a different source  c) no description of the derivation of the non exposed cohort  3) Ascertainment of exposure  a) secure record (e.g. surgical records) *  b) structured interview *  c) written self report  d) no description  4) Demonstration that outcome of interest was not present at start of study  a) yes *  b) no  ***Comparability***  1) Comparability of cohorts on the basis of the design or analysis  a) study controls for Injury severity *  b) study controls for any additional factor * (Age, gender, type of mechanism, altered level of consciousness, admission to ICU)  ***Outcome***  1) Assessment of outcome  a) independent blind assessment *  b) record linkage *  c) self report  d) no description  2) Was follow-up long enough for outcomes to occur  a) yes (select an adequate follow up period for outcome of interest) *  b) no  3) Adequacy of follow up of cohorts  a) complete follow up - all subjects accounted for *  b) subjects lost to follow up unlikely to introduce bias - small number lost - > <20% loss to follow up, or description provided of those lost) *  c) follow up rate <80% and no description of those lost  d) no statement |
| --- |

*appropriate method of assessment (equates to a score of 0). If any of the parameters are assessed using a method without asterisk, a score of 1 point is attributed. The total score reflects risk of bias. 0-1: low, 2-3: moderate, >3: high.

Appendix 2 – Risk of Bias of included studies

| Author, year | Selection (max 4 items) | Comparability (max 1 item) | Outcome (max 3 items) | Comments |
| --- | --- | --- | --- | --- |
| Enderson, 1990 | 1 – no description of non-exposed cohort | 1 – no description of non-exposed cohort, just summary statistic given | 0 | Total score: 2 - Moderate risk |
| Biffl, 2003 | 0 | 0 – all demographic variables comparable in both cohorts | 0 | Total score: 0 - Low risk |
| Vles, 2003 | 1 – no description of non-exposed cohort | 1 – no description of non-exposed cohort | 0 | Total score: 2 - Moderate risk |
| Hoff, 2004 | 1 – no description of non-exposed cohort | 1 – no description of non-exposed cohort | 0 | Total score: 2 - Moderate risk |
| Soundappan, 2004 | 1 – no description of non-exposed cohort | 1 – no description of non-exposed cohort | 0 | Total score: 2 - Moderate risk |
| Howard, 2006 | 1 – no description of non-exposed cohort | 1 – no description of non-exposed cohort | 0 | Total score: 2 - Moderate risk |
| Okello, 2007 | 1 – no description of non-exposed cohort | 1 – no description of non-exposed cohort | 0 | Total score: 2 - Moderate risk |
| Janjua, 2008 | 1 – no description of non-exposed cohort | 1 – no description of non-exposed cohort | 0 | Total score: 2 - Moderate risk |
| Ursic, 2009 | 0 | 0 – all demographic variables comparable in both cohorts | 0 – No missed injury description in article. Obtained through author communication | Total score: 0 - Low risk |
| Huynh, 2010 | 1 – no description of non-exposed cohort | 1 – no description of non-exposed cohort | 0 | Total score: 2 - Moderate risk |

Risk of Bias using Newcastle Ottawa Scale.^20^ The total score reflects risk of bias. 0-1: low, 2-3: moderate, >3: high.
